# Supplementary material for: Elastic conducting polymer composites in thermoelectric modules
Source: Nat Commun. 2020 Mar 18;11:1424. doi: 10.1038/s41467-020-15135-w (PMC7080746; doi:10.1038/s41467-020-15135-w)
Supplement: Supplementary file 1 — Supplementary Information [file 41467_2020_15135_MOESM1_ESM.pdf]

Supplementary Information

Elastic Conducting Polymer Composites in Thermoelectric Modules

N. Kim et al.

Contents:

Supplementary Figures 1-20
Supplementary Tables 1-2
Supplementary References

Supplementary Figures

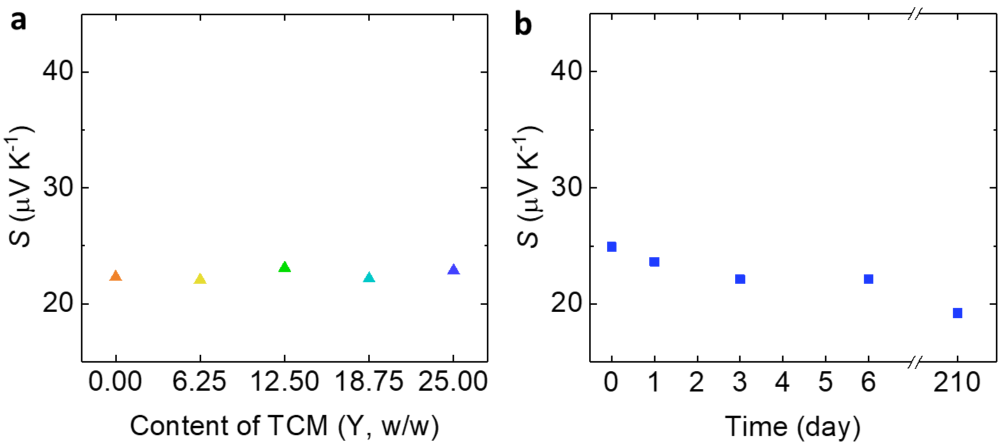

Supplementary Figure 1| Seebeck coefficients of CP films. (a) *S* of CP films with varying content of TCM (Y). The relative content of CP is 15. 0.15 wt% of an ammonia solution was added with respect to the CP dispersion as in the case for the composite with WPU. (b) *S* of the optimized composite (CP:TCM:WPU=15:25:85, *w*/*w*) as a function of storage time in ambient conditions.

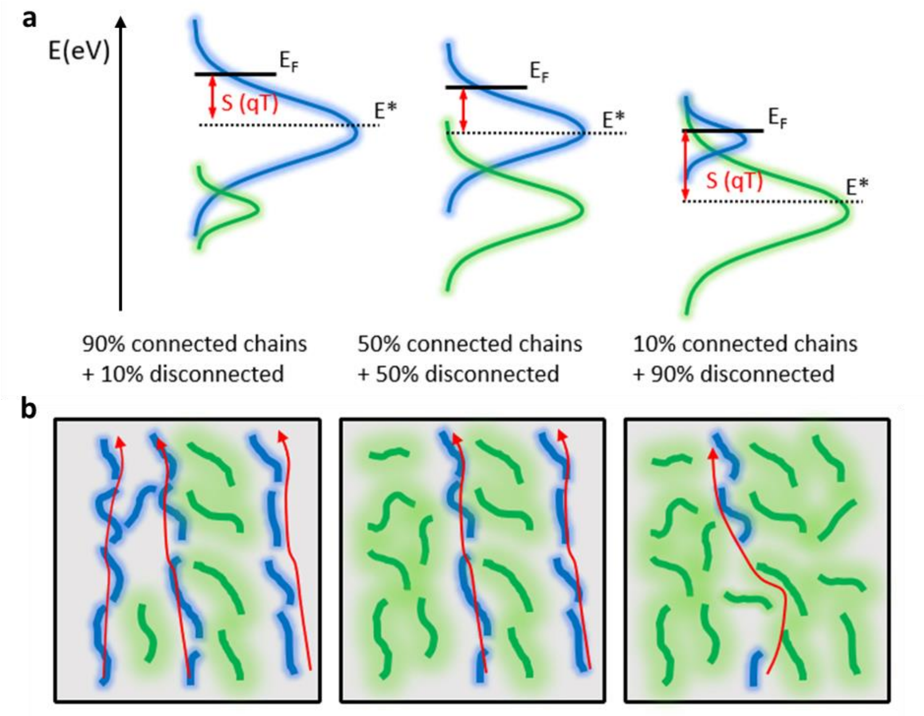

Supplementary Figure 2| Hypothesis to explain a strong correlation between *S* and σ_{dc}. Schematic drawings of different regimes of PEDOT connectivity (b) and the corresponding electronic energy structures (a).

In order to explain the correlation between σ_{dc} and *S* shown in Fig. 2c & 2f, we propose the following hypothesis: In good approximation, the transport of charge carriers in a hopping regime between localized states is governed by the density of state (DOS). The Fermi distribution dictates the filling of the DOS; which also determines a level of energy called the transport energy E^*[1], dominating the percolation transport because of its highest hopping probability. The Seebeck coefficient (*S*) can be described as[2,3]:

$$S \cong -\frac{E^* - E_F}{qT}$$

, where q is the charge of the carrier, T the temperature, E_F is the Fermi Level.

The density of percolation paths in PEDOT:PSS is strongly affected by the morphology of the film as known by the addition of high boiling point solvents and ionic liquids that promotes the demixing of the excess of insulating PSS and promotes the creation of 3D percolation paths[4,5]; or by the addition of insulating polymers breaking the percolation paths[6]. Sometime small amounts of those additives lead to a drastic change in transport properties. For instance, the same absorption spectrum is observed for films with an electrical conductivity varying by 3 orders of magnitude[7], thus indicating that the concentration of PEDOT chains is barely affected, but their connectivity is much different and governed by microscopic morphology. Hence, we can thus speak about PEDOT chains with good connectivity and bad connectivity. PEDOT chains with good connectivity can form nanocrystals where short range order dominates the transport (blue domains in Supplementary Fig. 2). While PEDOT chains with bad connectivity are in an amorphous phase without even local order (green domains). The electrostatic potential profile on those two different types of PEDOT chains is different[8] and it is thus expected to affect their electronic energy levels. We propose that their DOS are thus also different. As a result, our hypothesis is to consider a DOS for PEDOT:PSS systems composed two DOS peaks. One DOS peak representing the electronic levels for the connected PEDOT chains and another DOS peak representing the electronic bipolaronic levels of the disconnected PEDOT chains. In this hypothesis, we thus propose that upon the removal of ionic liquid or the addition of insulating WPU polymer, the ratio of connected *vs.* disconnected PEDOT chains decreases and the intensity ratio of those two DOS peaks differs, which leads to an increase of S as proposed recently for polymer blends[9].

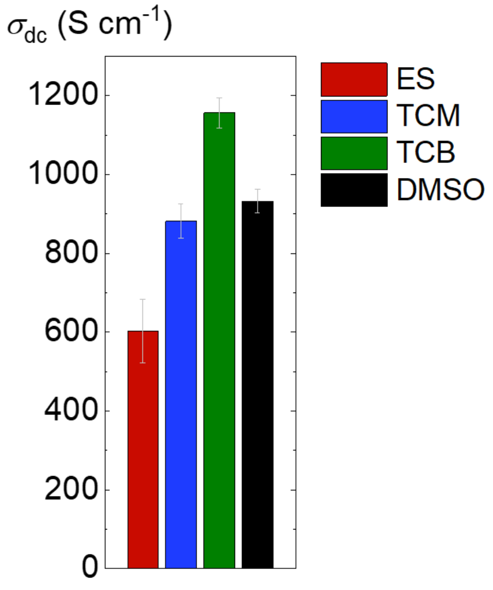

Supplementary Figure 3| σ_{dc} of CP films with a different type of ILs or DMSO. Composition ratio, CP:IL=12:20, *w*/*w*. For the CP-DMSO films, 5.6 wt% of DMSO was added to the CP solution dispersion. The average σ_{dc} of CP-TCM films is 880 S cm^{-1}, which translates in an σ_{dc} for the CP part of approximately 2350 S cm^{-1} considering the mass fraction of CP in the CP-TCM film.

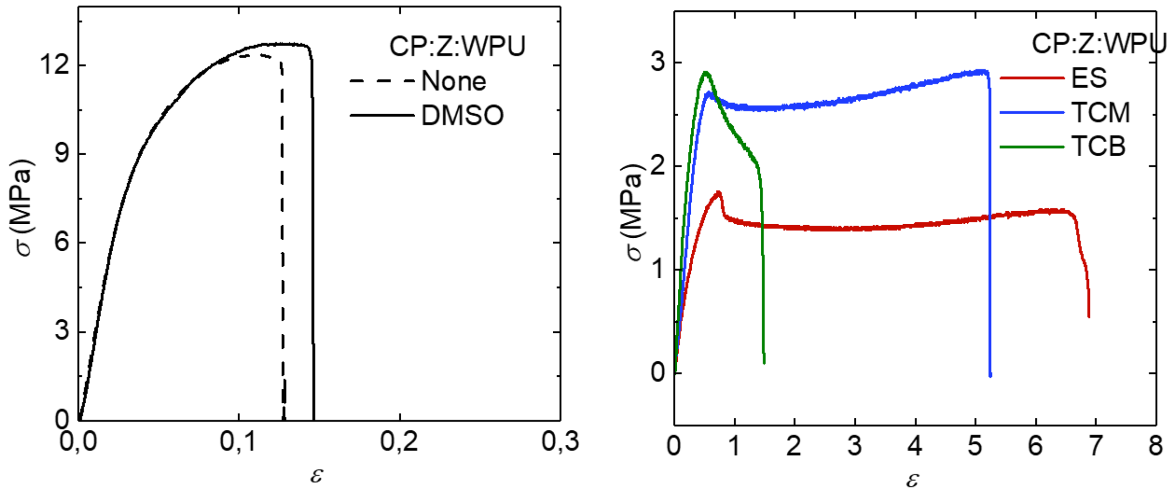

Supplementary Figure 4| Stress-strain curves of CP-WPU composites. With and without different types of ILs or DMSO. Composition ratio, CP:WPU=15:85, CP:IL:WPU=15:25:85, *w*/*w*. For the composite with DMSO, 5.6 wt% of DMSO was added to the CP dispersion before making the composite.

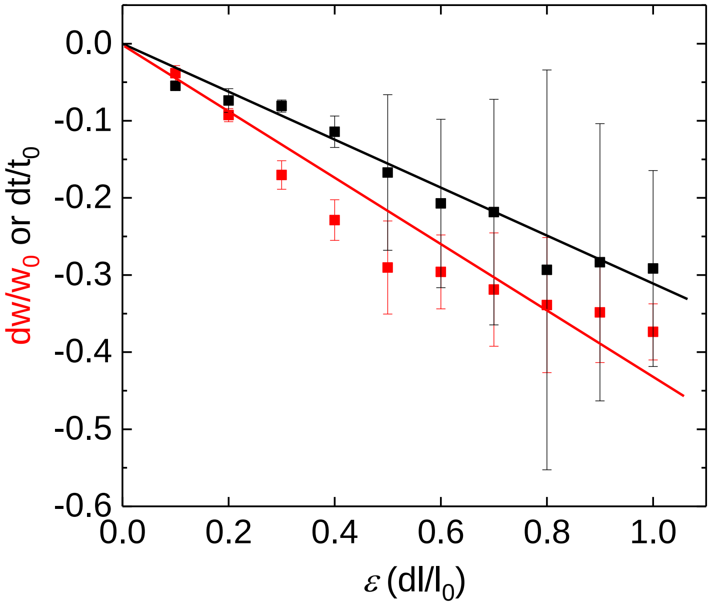

Supplementary Figure 5| Geometrical shape changes of composites under tensile strain. dl/l_0, length change. Composition ratio, CP:TCM:WPU=15:25:85, *w/w*. Linear lines indicate dw/w_0 (width change) and dt/t_0 (thickness change) for Poisson's ratios of 0.43 and 0.3, respectively. Error bars represent the SD. R_G/R_0 (*exp.*) in Fig. 3b was estimated from the average values measured for dw/w_0 and dt/t_0 plotted in Supplementary Fig. 5 according to the relation:

$$\frac{R_G}{R_0} = \frac{1 + dl/l_0}{(1 + dw/w_0)(1 + dt/t_0)}$$

, while R_G/R_0 for Poisson's ratio 0.5 is calculated from the equation:

$$\frac{R_G}{R_0} = \frac{1 + \nu}{(1 - 0.5\nu)(1 - 0.5\nu)}$$

, where ν is the tensile strain which is the same as dl/l_0.

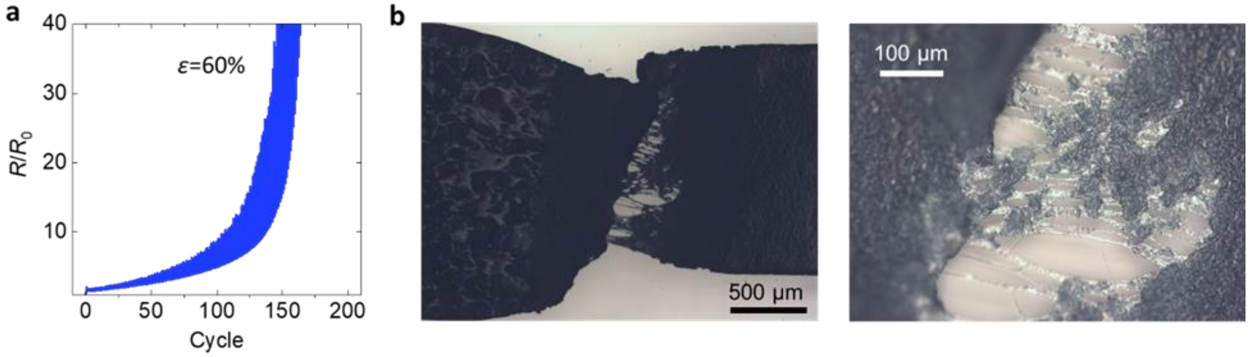

Supplementary Figure 6| R/R_0 under cyclic strain at 60%. (a) R/R_0 of the free-standing optimized composite (CP:TCM:WPU=15:25:85, *w*/*w*) under cyclic strain at 60%. (b) Optical microscope images of the torn parts of the composite after 200 cycles.

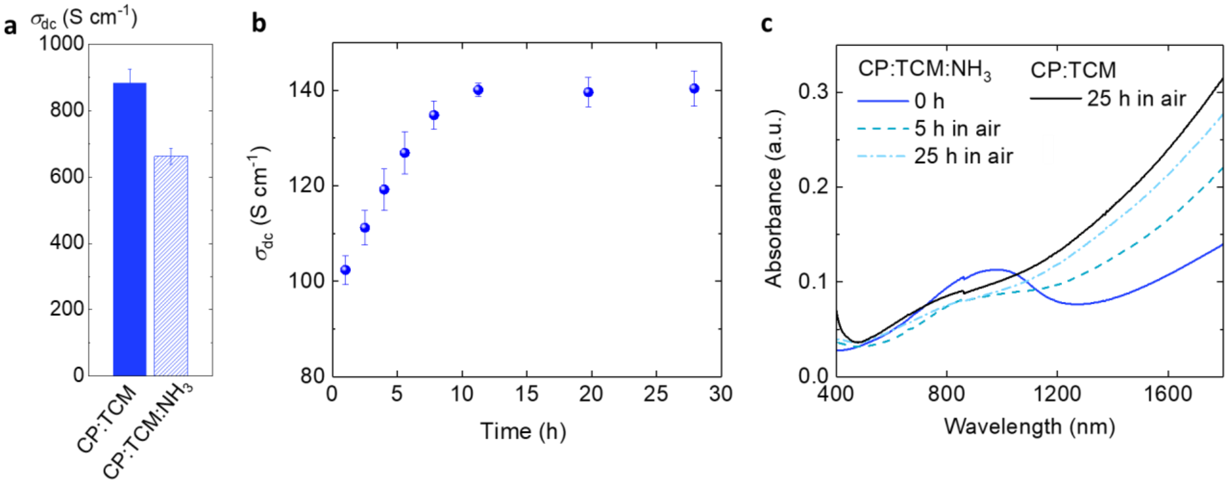

Supplementary Figure 7| Effect of adding ammonia on σ_{dc} of CP-TCM(-WPU) films. (a) Comparison of σ_{dc} of CP-TCM films with and without the addition of ammonia (NH_3). Composition ratio, CP:TCM=12:20 or CP:TCM:NH_3=12:20:1.5, *w*/*w*. Error bars represent the SD.

(b) Change in σ_{dc} of CP-TCM-WPU films over time. Composition ratio, CP:TCM:WPU=15:25:85, *w/w*. Error bars represent the SD. (c) Comparison of absorbance spectra of CP-TCM films over time. The films were stored in ambient air. The CP-TCM film coated from the dispersion containing NH_3 initially shows a pronounced polaron peak at around 900 nm and a reduced free carrier absorption in the NIR region[10] as a result of partial dedoping of PEDOT. As the CP-TCM-NH_3 film was stored in air, the NIR absorption increased with a decrease in the polaron peak and the spectrum became identical with that of the CP-TCM film coated from the dispersion not containing NH_3 after 25 hours of storage in air. Composition ratio, CP:TCM:NH_3=12:20:1.5 or CP:TCM=12:20, *w/w*.

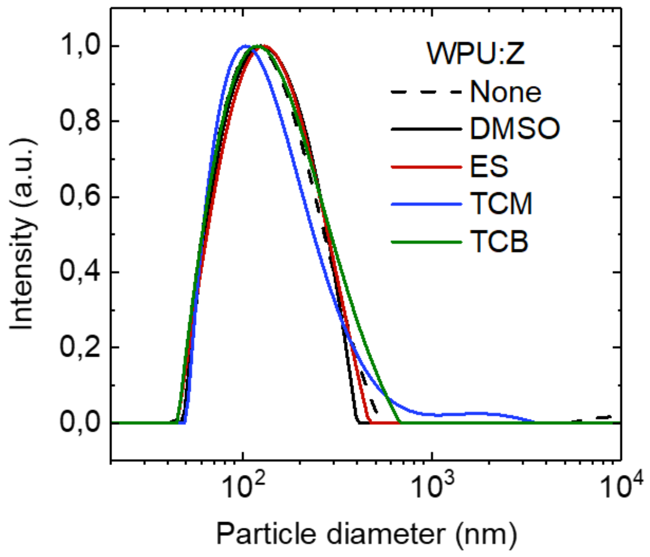

Supplementary Figure 8| DLS studies on particle size distribution of WPU dispersions. With and without a different type of ILs or DMSO. Composition ratio, WPU:IL=85:25, WPU:DMSO=85:70, *w/w*.

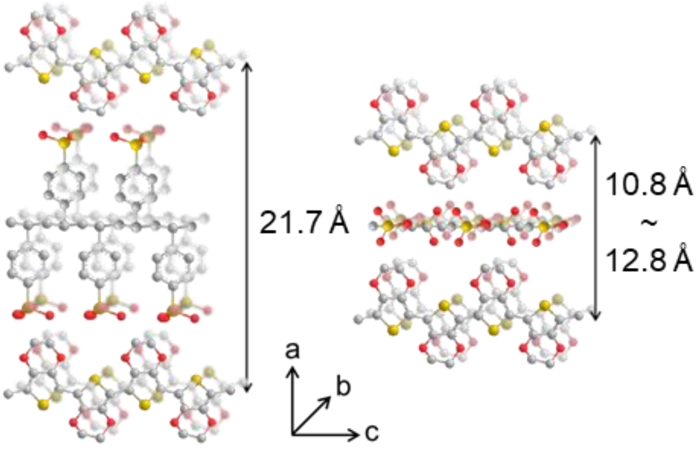

Supplementary Figure 9| Molecular packing structures of PEDOT:PSS. The lattice parameters, a, b, and c, correspond to the lamellar stacking distance $d_{(100)}$ of alternate orderings of PEDOT and PSS, the π-π stacking distance $d_{(010)}$ of PEDOT aromatic rings, and the repeat distance of two EDOT units, respectively. Reproduced with permission from Kee, S. *et al. Adv. Mater.* **28**, 8625-8631 (2016), Copyright 2016 WILEY-VCH Verlag GmbH & Co. KGaA, Weinheim.

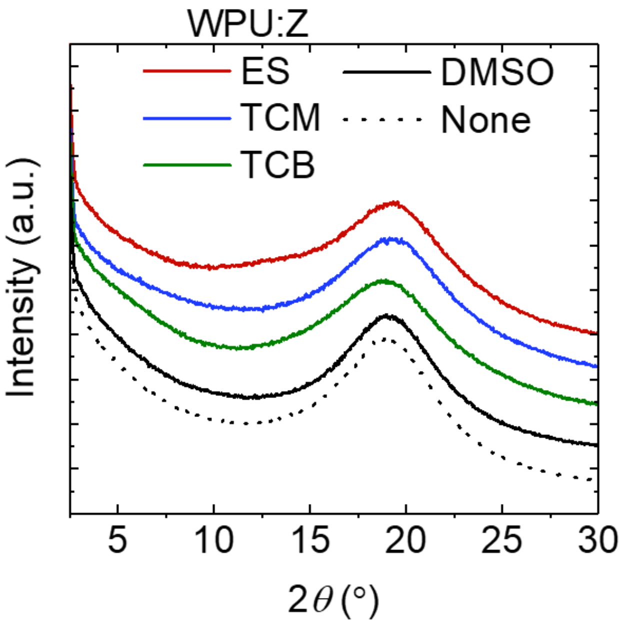

Supplementary Figure 10| XRD patterns of WPU films. With and without a different type of ILs or DMSO. Composition ratio, WPU:IL=85:25, *w*/*w*. Composition ratio in a WPU-DMSO dispersion before making films, WPU:DMSO=85:70, *w*/*w*.

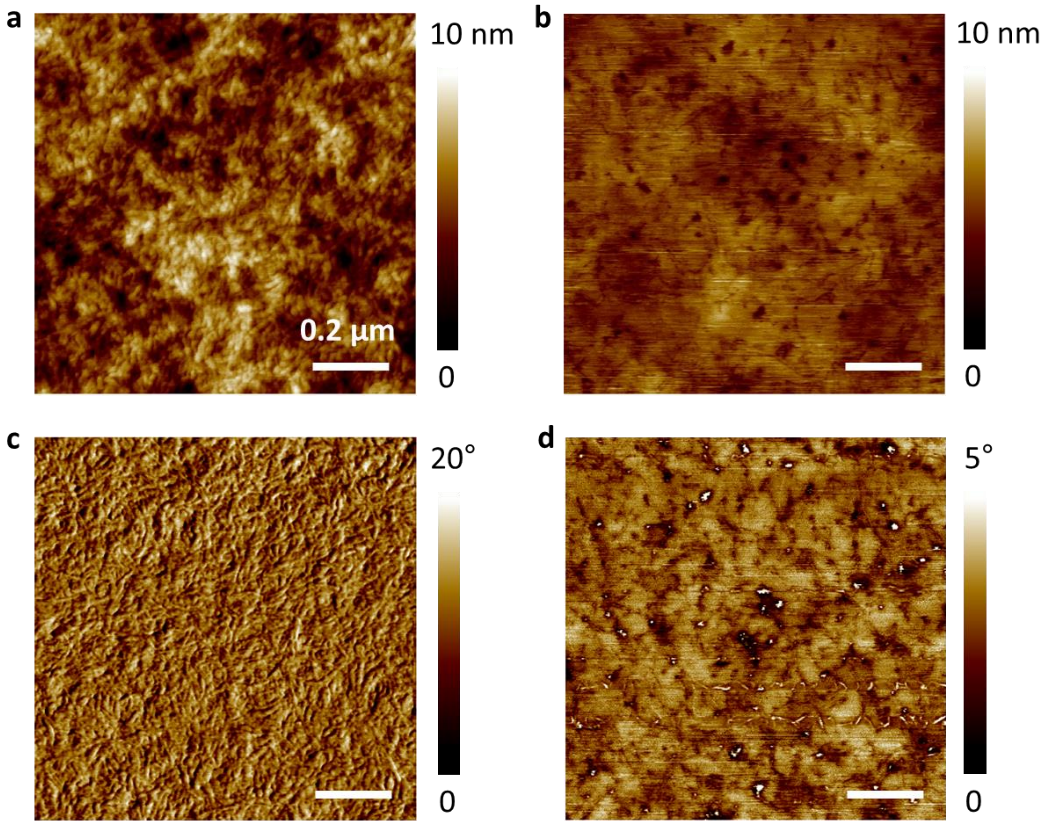

Supplementary Figure 11| AFM images of CP films. AFM height (a & b) and phase (c & d) images of CP films with (b & d) and without (a & c) TCM. The images were taken from the samples without a post-washing process. Scale bars, 0.2 µm.

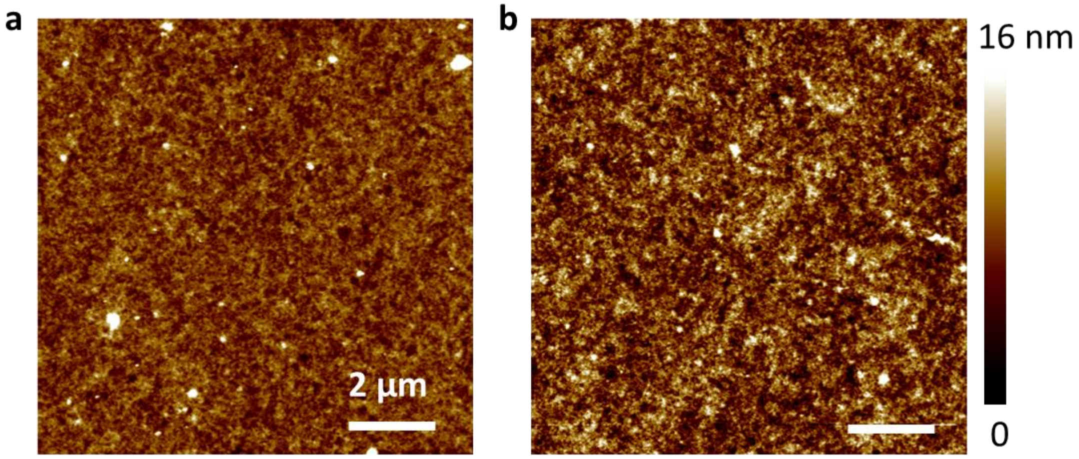

Supplementary Figure 12| Larger-scale AFM height images of CP films. With (b) and without (a) TCM. The images were taken after a post-washing process. Scale bars, 2 µm.

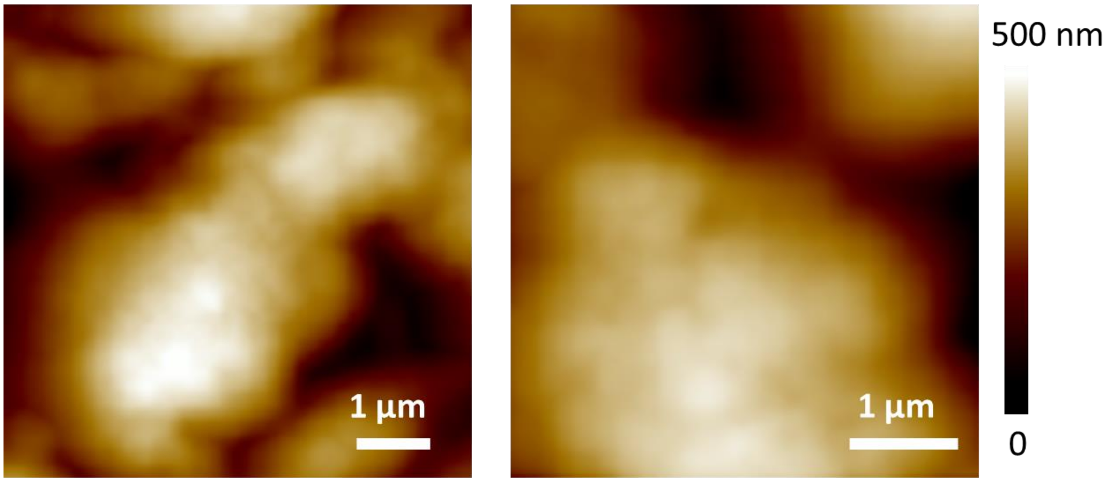

Supplementary Figure 13| Smaller-scale AFM height images of a CP-WPU composite film. Scale bars, 1 µm.

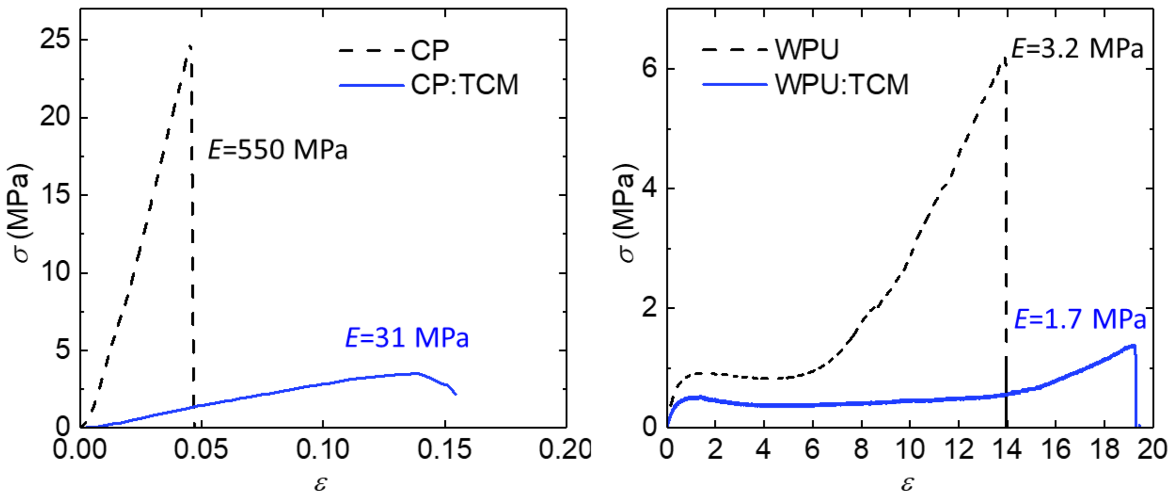

Supplementary Figure 14| Stress-strain curves of CP and WPU. With and without TCM. Young's modulus values estimated from initial linear slopes of stress-strain curves are shown next to each curve. Composition ratio, CP:TCM=15:25, WPU:TCM=85:25, *w*/*w*.

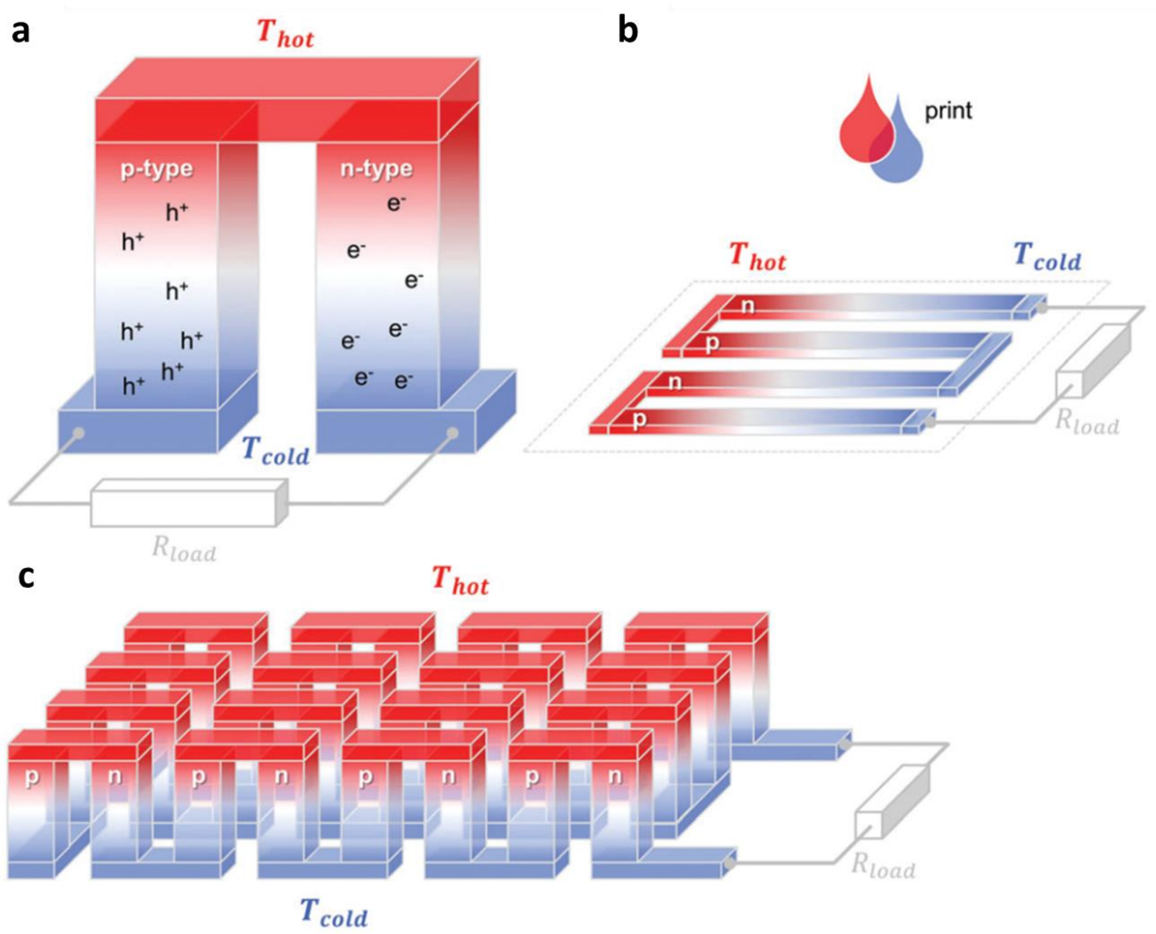

Supplementary Figure 15| TE device configurations. (a) Vertical and (b) lateral TE device configurations composed of one *p*-type and one *n*-type TE legs. (c) A vertical thermoelectric module composed of multiple *p*-type and *n*-type TE legs connected electrically in series and thermally in parallel. Reproduced from Kroon, R. *et al. Chem. Soc. Rev.* **45**, 6147-6164 (2016) with permission of The Royal Society of Chemistry.

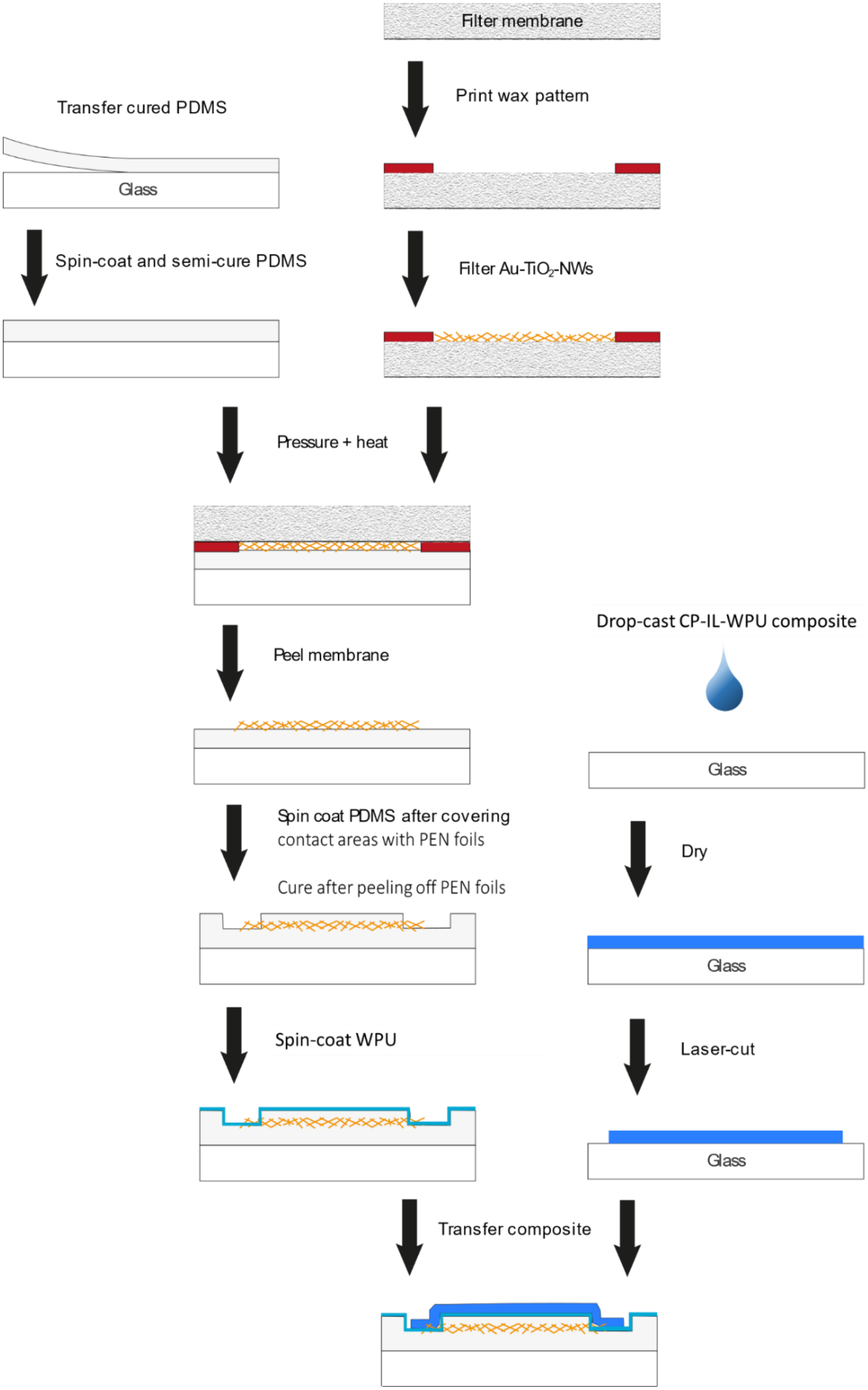
Filter membrane
Print wax pattern
Transfer cured PDMS
Glass
Spin-coat and semi-cure PDMS
Filter Au-TiO2-NWs
Pressure + heat
Drop-cast CP-IL-WPU composite
Peel membrane
Glass
Spin coat PDMS after covering
contact areas with PEN foils
Cure after peeling off PEN foils
Dry
Glass
Spin-coat WPU
Laser-cut
Glass
Transfer composite

Supplementary Figure 16| Fabrication of a stretchable thermoelectric module. Processing schematics following the arrows.

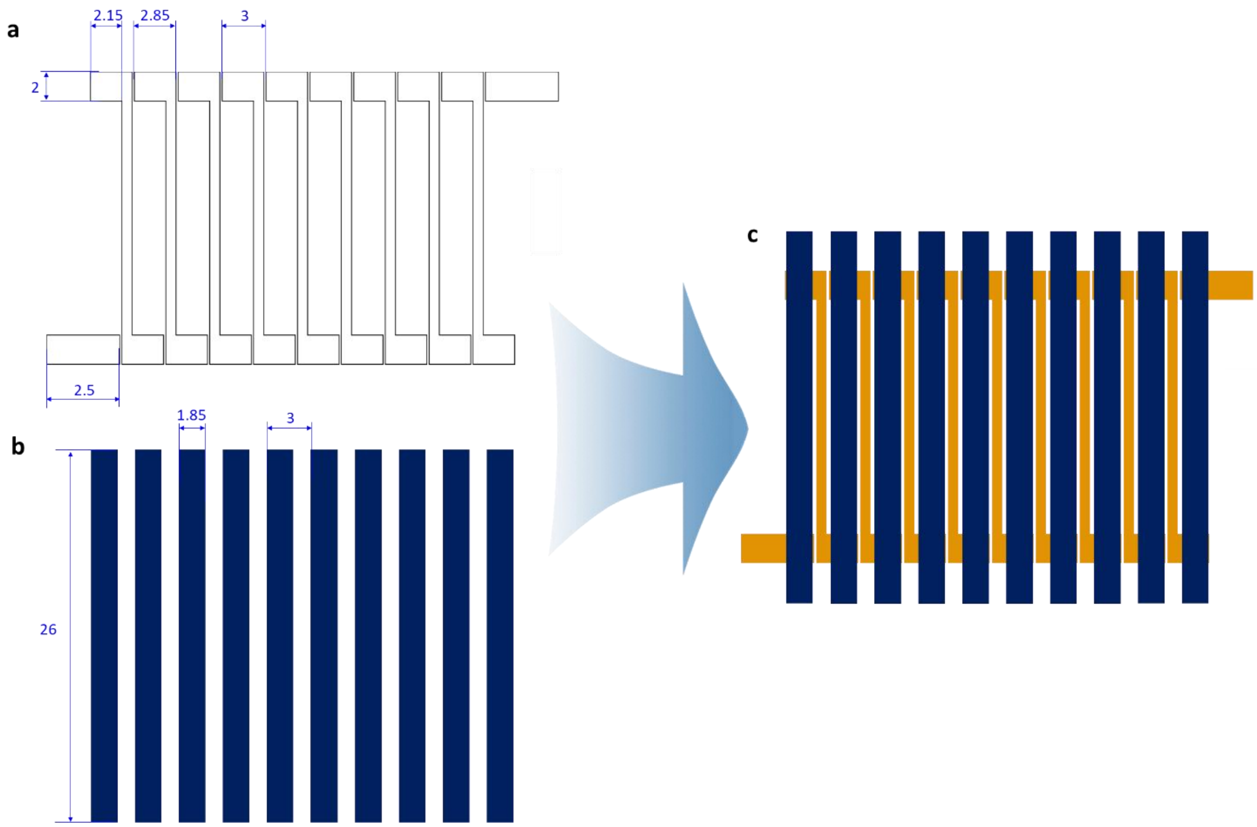

Supplementary Figure 17| Dimension of a stretchable thermoelectric module. (a) Au-TiO_2 NW interconnects. (b) Composite TE legs. (c) A TE module. Scales in mm.

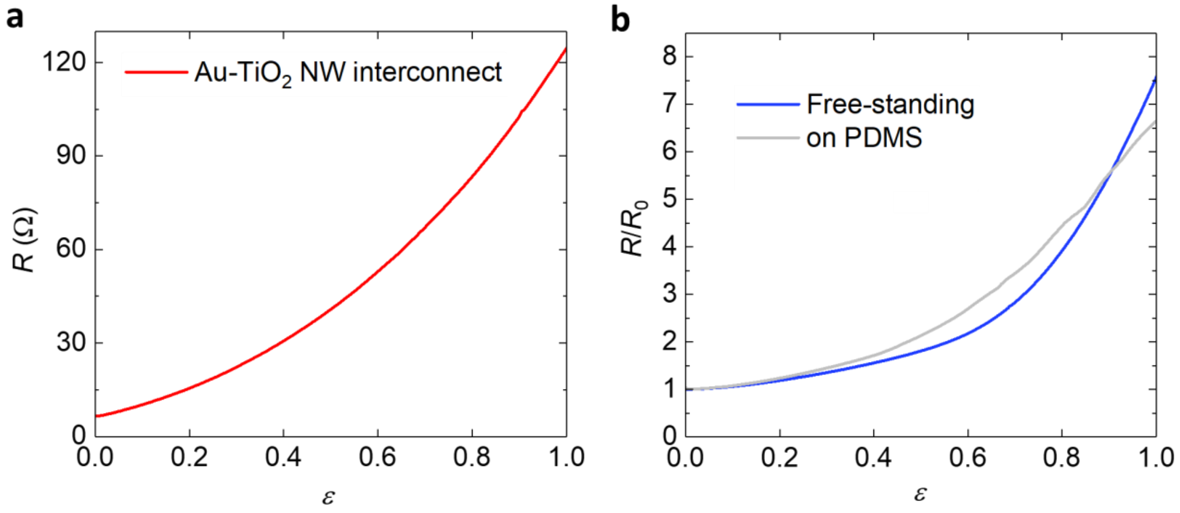

Supplementary Figure 18| Resistance *vs*. strain of an interconnect and a composite TE leg. Resistance of (a) an Au-TiO_2 NW interconnect embedded in a PDMS substrate and (b) a TE leg (i.e. the optimized composite film supported on a PDMS substrate, gray line) as a function of tensile strain with the comparison to the free-standing optimized composite film (data from Fig. 3b, blue line). Different electromechanical properties with and without PDMS support can originate from different mechanical deformations between the composite and a PDMS substrate since the composite (~40 μm) supported on a PDMS substrate conforms to the substrate (>200 μm) when deformed. Nonetheless, the difference is not significant under tensile strain up to 40%.

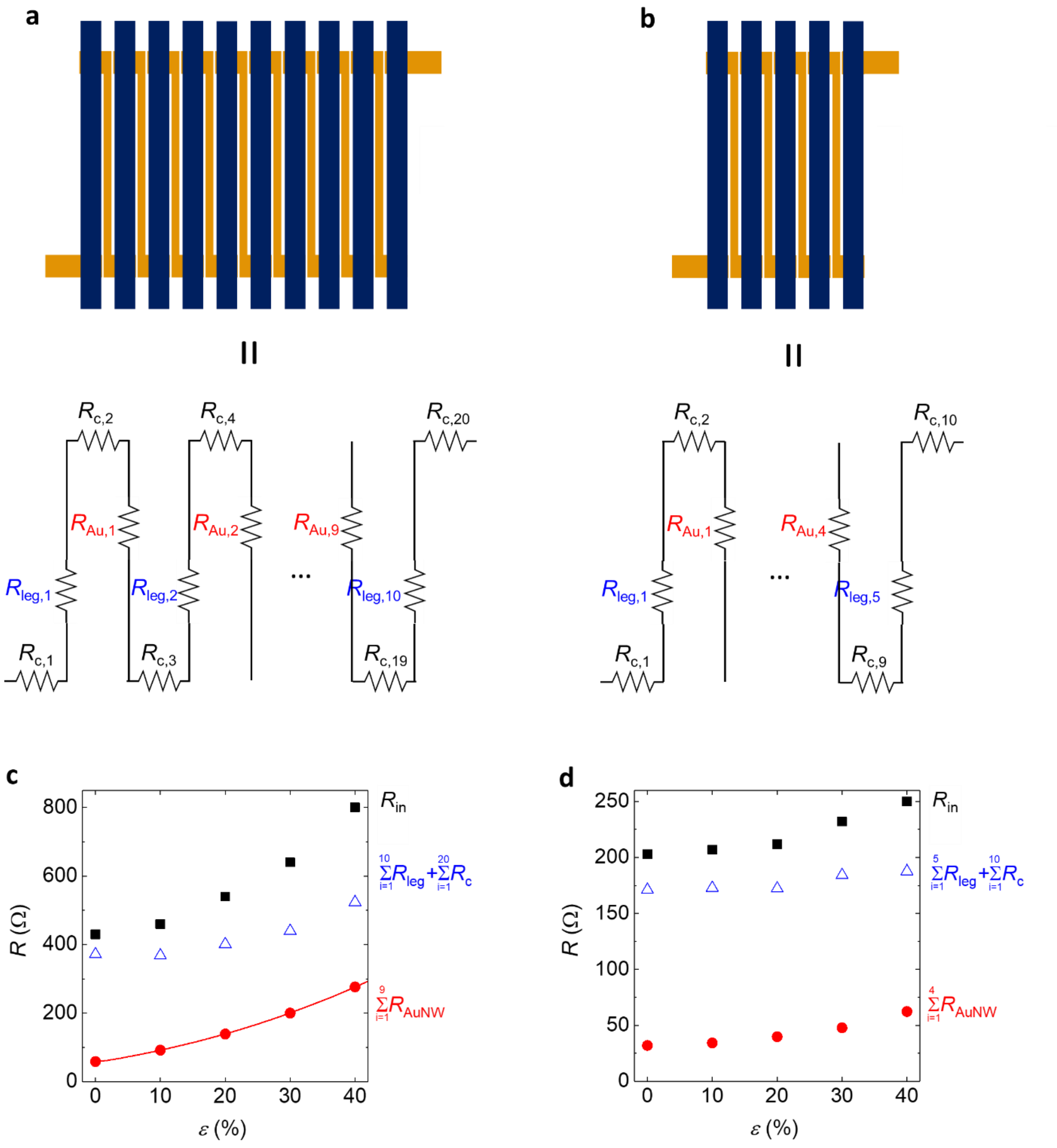

Supplementary Figure 19| Analysis of strain-dependent resistances with equivalent circuits. Schematics of TE modules characterized in Fig. 6 under tensile strain in the direction (a) parallel with and (b) perpendicular to thermodiffusion. Equivalent circuits to calculate the internal resistance of TE modules (R_{in}) were drawn below. $R_{in}=\Sigma R_{leg}+\Sigma R_{Au}+\Sigma R_c$, where R_{leg}, R_{Au}, and R_c

are the resistances of a TE leg, an Au-TiO_2 NW interconnect, and a contact between a leg and an interconnect, respectively. (c & d) Contributions of ΣR_{Au} and ΣR_{leg}+ΣR_c to R_{in}. ΣR_{Au} was estimated by multiplying R of a single interconnect measured under different tensile strain and the number of interconnects in each module (i.e. 9 for (c) and 4 for (d)). R_{Au} *vs.* strain (ε) in (c) was obtained on a motorized linear stage with gold-coated 4-point contact pads, while R_{Au} *vs.* ε in (d) was measured in the same way as for the TE module by the 2-point probe method. ΣR_{leg}+ΣR_c *vs.* ε was estimated by subtracting ΣR_{Au} from R_{in}. Comparing the changes in R_{in} and ΣR_{Au} reveals that the R_{in} increase arose mainly from the increase in ΣR_{Au}, upon both parallel and perpendicular stretching, while the change in ΣR_{leg}+ΣR_c has minor contribution to the R_{in} increase. Despite a reduced length of interconnects upon perpendicular stretching, the ΣR_{Au} increase can be caused by the reduced density of connections between conductive NWs.

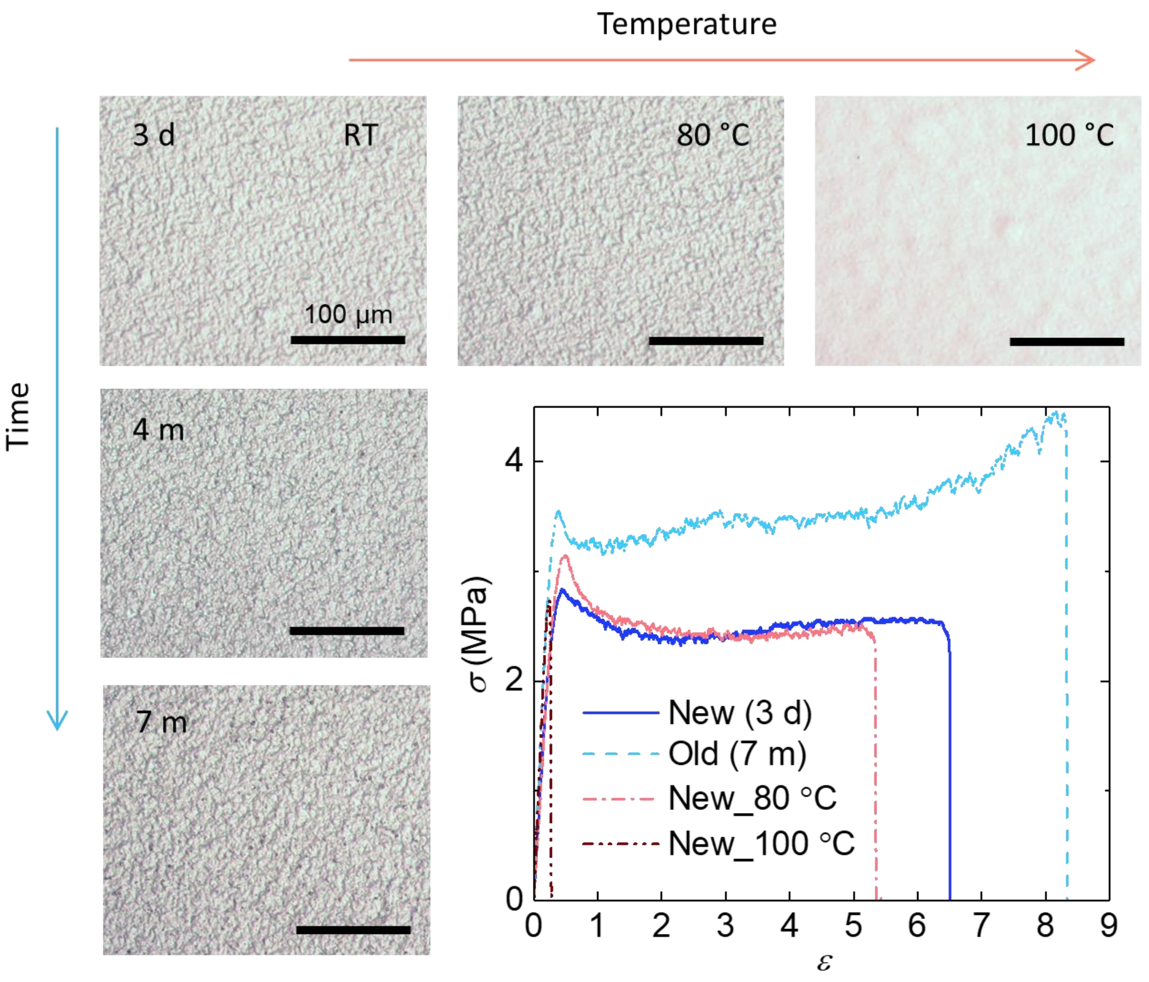

Supplementary Figure 20| Stability of the composite in air and under thermal annealing. Optical microscope images and stress (σ)-strain (ε) curves of the optimized composite (CP:TCM:WPU=15:25:85, *w*/*w*) films used for TE legs after storage time up to 7 months in ambient conditions or after heating up to 100 °C. The similar surface morphology and mechanical property preserved after 7 months storage time or thermal annealing at 80 °C show the excellent stability of the composite with a stable dispersion of ionic liquid plasticizers. Considering the preserved electrical and mechanical properties over time, the tiny lumps observed on the surface of old samples may result from the migration of small-molecular polyurethanes. Thermal annealing at 100 °C led to a significantly different morphology in conjunction with a severe deterioration in

mechanical property (i.e. elongation at break decreased down to 27%). Based on haze in the image and poor mechanical property observed for the composite without ionic liquids, we assume that the phase separation between different components and the migration of ionic liquids to the surface may occur at this temperature.

Supplementary Tables

Supplementary Table 1| Various PEDOT- or PEDOT:PSS-elastomer composites. Compositions, fabrication procedures, and their performances as elastic conductors were compared.

Conductive Filler	Elastomer	Content of Elastomer	Procedure	Solvent	Electrical Conductivity	Fracture Strain	Elastic Recovery	Modulus	Ref.
PEDOT:PSS	WPU	85 wt%	Aqueous solution blending	Water, IL	140 ± 5 S/cm	500 ± 130%	75% (after 200% stretch)	6.9 ± 1.7 MPa	This work
PEDOT:PSS	WPU	86 wt%	Aqueous solution blending	Water, DMSO	48 S/cm	N/A	N/A	N/A	[11]
PEDOT:PSS	WPU	95 wt%	Aqueous solution blending	Water, DMSO	14.96 S/cm	217 ± 5%	N/A	23.93 ± 0.84 MPa	[12]
PEDOT:PSS (5 wt%) /rGO (1 wt%)	WPU	94 wt%	Aqueous solution blending	Water, DMSO	18.2 S/cm	530%	83% (after 30% stretch)	246.2 MPa	[13]
PEDOT	PU	50 wt%	Spin-coat PU/EDOT/Fe(III)(TsO)3 mixture on Si substrate, polymerize at 65 °C, thoroughly rinse with water	THF, anisole, butanol	120 S/cm	N/A	N/A	N/A	[14]
PEDOT	PU	80 wt%	Spin-coat PU/EDOT/ Fe(III)(TsO)3 mixture on glass, polymerize at 100 °C, thoroughly rinse with water	THF, butanol	10 S/cm	270%	N/A	14 MPa	[15]
PEDOT:PSS	PU	87 wt%	Disperse PU in DMSO, solution blending, fiber spinning	Water, DMSO	9.39 ± 0.42 S/cm	345.2 ± 15.2%	72.2% (after 200% stretch)	23.47 ± 2.16 MPa	[16]
PEDOT:PSS	Spandex yarn (kind of PU)	90 wt%	Freeze-dry PEDOT:PSS, re-disperse and homogenize in DMSO, disperse Lycra yarn in DMSO, then, solution blending<br>*EG treatment: after drop-cast and dry the film at 80'C, immerse the film in an EG bath, then dry	DMSO, EG	62 ± 4 S/cm (79 ± 5 S/cm after EG treat.)	300 ± 230% (700 ± 150% after EG treat.)	N/A	23 ± 11 MPa (75 ± 65 MPa after EG treat.)	[17]
PEDOT:PSS	PDMS	N/A	Freeze-dry PEDOT:PSS, infiltrate PDMS oligomer/curing agent/hexane, then, cure at 80 °C	Hexane	5 S/cm	52%	N/A	N/A	[18]
PEDOT:PSS	PDMS, PDMS-b-PEO	39 wt%	Blend PDMS oligomer/curing agent/PDMS-b-PEO/PEDOT:PSS, then, cure	Water	0.26–0.38 S/cm	75%	N/A	395 kPa	[19]
PEDOT:PSS	P(BA–St) latexes	83 wt%	Solution blending with surfactant (DBSA)	Water, DMSO	63 S/cm	97%	N/A	N/A	[20]
PEDOT:PIL	poly(ether-b-ester)	99 wt%	Polymerize EDOT in PIL aqueous solution, phase transfer reaction with hydrophobic anion, ground the precipitated powder, re-disperse in PC, blend with PEEA	Propylene carbonate (PC)	N/A	350%	N/A	N/A	[21]

Supplementary Table 2| Quantitative analysis of AFM height images. Lateral cluster diameter and surface roughness of CP-WPU composite films with and without a different type of ILs or DMSO. The cluster diameter was averaged from the five biggest clusters shown in Fig. 5b, while the surface roughness was determined from the RMS values taken from three 20×20 μm^2 height images for each sample.

Sample	Lateral cluster diameter (μm)	RMS surface roughness (nm)
CP-WPU	3.06 ± 0.19	126 ± 8
CP-DMSO-WPU	2.61 ± 0.25	138 ± 8
CP-ES-WPU	1.16 ± 0.08	67 ± 7
CP-TCM-WPU	1.50 ± 0.14	42 ± 5
CP-TCB-WPU	1.91 ± 0.23	62 ± 2

Supplementary References

[1] Baranovskii, S. D., Faber, T., Hensel, F. & Thomas, P. The applicability of the transport-energy concept to various disordered materials. *J. Phys.: Condens. Matter* **9**, 2699-2706 (1997).

[2] Schmechel, R. Hopping transport in doped organic semiconductors: A theoretical approach and its application to p-doped zinc-phthalocyanine. *J. Appl. Phys.* **93**, 4653-4660 (2003).

[3] Ihnatsenka, S., Crispin, X. & Zozoulenko, I. V. Understanding hopping transport and thermoelectric properties of conducting polymers. *Phys. Rev. B* **92**, 035201 (2015).

[4] Kee, S. *et al.* Controlling Molecular Ordering in Aqueous Conducting Polymers Using Ionic Liquids. *Adv. Mater.* **28**, 8625-8631 (2016).

[5] Shi, H., Liu, C., Jiang, Q. & Xu, J. Effective Approaches to Improve the Electrical Conductivity of PEDOT:PSS: A Review. *Adv. Electron. Mater.* **1**, 1500017 (2015).

[6] Seyedin, M. Z., Razal, J. M., Innis, P. C. & Wallace, G. G. Strain-Responsive Polyurethane/PEDOT:PSS Elastomeric Composite Fibers with High Electrical Conductivity. *Adv. Funct. Mater.* **24**, 2957-2966 (2014).

[7] Wijeratne, K. *et al.* Bulk electronic transport impacts on electron transfer at conducting polymer electrode–electrolyte interfaces. *Proc. Natl. Acad. Sci.* **115**, 11899-11904 (2018).

[8] Muñoz, W. A., Crispin, X., Fahlman, M. & Zozoulenko, I. V. Understanding the Impact of Film Disorder and Local Surface Potential in Ultraviolet Photoelectron Spectroscopy of PEDOT. *Macromol. Rapid Commun.* **39**, 1700533 (2018).

[9] Zuo, G., Abdalla, H. & Kemerink, M. Conjugated Polymer Blends for Organic Thermoelectrics. *Adv. Electron. Mater.* **5**, 1800821 (2019).

[10] Massonnet, N. *et al.* Improvement of the Seebeck coefficient of PEDOT:PSS by chemical reduction combined with a novel method for its transfer using free-standing thin films. *J. Mater. Chem. C* **2**, 1278-1283 (2014).

[11] Choong, C.-L. *et al.* Highly Stretchable Resistive Pressure Sensors Using a Conductive Elastomeric Composite on a Micropyramid Array. *Adv. Mater.* **26**, 3451–3458 (2014).

[12] Li, P. *et al.* Stretchable and conductive polymer films for high-performance electromagnetic interference shielding. *J. Mater. Chem. C*, **4**, 6525–6532 (2016).

[13] Zhou, R. *et al.* Stretchable heaters with composites of an intrinsically conductive polymer, reduced graphene oxide and an elastomer for wearable thermotherapy. *J. Mater. Chem. C*, **5**, 1544–1551 (2017).

[14] Hansen, T. S. *et al.* Highly Stretchable and Conductive Polymer Material Made from Poly(3,4-ethylenedioxythiophene) and Polyurethane Elastomers. *Adv. Funct. Mater.* **17**, 3069–3073 (2007).

[15] Kai, H. *et al.* Intrinsically Stretchable Electrochromic Display by a Composite Film of Poly(3,4-ethylenedioxythiophene) and Polyurethane. *ACS Appl. Mater. Interfaces* **9**, 19513−19518 (2017).

[16] Seyedin, M. Z. *et al.* Strain-Responsive Polyurethane/PEDOT:PSS Elastomeric Composite Fibers with High Electrical Conductivity. *Adv. Funct. Mater.* **24**, 2957–2966 (2014).

[17] Taroni, P. J. *et al.* Toward Stretchable Self-Powered Sensors Based on the Thermoelectric Response of PEDOT:PSS/Polyurethane Blends. *Adv. Funct. Mater.* **28**, 1704285 (2018).

[18] Teng, C. *et al.* Polymer *in situ* embedding for highly flexible, stretchable and water stable PEDOT:PSS composite conductors. *RSC Adv.* **3**, 7219–7223 (2013).

[19] Noh, J.-S. Highly conductive and stretchable poly(dimethylsiloxane):poly(3,4-ethylenedioxythiophene):poly(styrene sulfonic acid) blends for organic interconnects. *RSC Adv.* **4**, 1857–1863 (2014).

[20] Yin, H.-E. *et al.* Innovative elastic and flexible conductive PEDOT:PSS composite films prepared by introducing soft latexes. *J. Mater. Chem.* **22**, 3800–3810 (2012).

[21] Kwon, S. J. *et al.* Elastomeric conducting polymer nano-composites derived from ionic liquid polymer stabilized-poly(3,4-ethylenedioxythiophene). *Synth. Met.* **160**, 1092–1096 (2010).
